# Impact of urban blue spaces on urban surface temperatures - A seasonal perspective

**DOI:** 10.1038/s41598-026-49643-4

**Published:** 2026-05-09

**Authors:** Lukas Fricke, Nadja Kabisch

**Affiliations:** Institute of Earth System Sciences, Physical Geography and Landscape Ecology Section, Leibniz University Hannover, Schneiderberg 50, 50167 Hannover, Germany

**Keywords:** Urban blue space, Remote Sensing, Urban planning, Climate sciences, Ecology, Ecology, Environmental sciences

## Abstract

Assessments on the cooling potential of natural environments in urban areas has largely focussed on green spaces at very specific spatial and temporal dimensions. Less has been studied so far about the surface thermal heat mitigation effect of urban blue spaces across an entire city and for different seasons and years. This study investigates the impact of urban blue spaces, such as lakes and ponds, on surface temperature for the city of Hannover, Germany. Using remote sensing data, we assess how different lake sizes and surrounding urban land use structures influence water surface temperature, surface temperature cooling intensity and distance across seasons and for three consecutive years. Our results indicate that larger lakes consistently exhibit the coolest water surface temperatures and provide the highest surface cooling intensities. The study identifies a case-specific Threshold Value of Efficiency (TVoE) of 0.70 ha for surface cooling intensity in the hottest months. We found that high proportions of impervious areas and buildings are associated with increased water surface temperatures and reduced surface temperature cooling effects, while high vegetation close to urban blue spaces show significantly lower surface temperature values. Given our limitations in data availability for particular environmental condition indicators (e.g. wind regime, air humidity) and water body characteristics (shape and depth) probably also influencing the thermal effect of blue spaces, we suggest a multi-method approach combining remote sensing and in-situ based environmental measurements to assess the thermal regulation of urban environments.

**Supplementary Information:**

The online version contains supplementary material available at 10.1038/s41598-026-49643-4.

## Introduction

Mitigating extreme heat presents a significant challenge for health and wellbeing in urban areas. Urban areas are often characterized by a high proportion of impervious areas and dense build-up environments, which exacerbate temperatures compared to surrounding rural regions through the phenomenon of the Urban Heat Island effect. Land surface temperatures in cities generally exceed those of surrounding areas, producing a pronounced Surface Urban Heat Island that often surpasses the air temperature–based Urban Heat Island^1,2^.

Many cities address the societal challenges of heat mitigation and the Urban Heat Island by the implementation of urban green-blue infrastructure elements^3^. Urban green infrastructure elements such as parks were shown to reduce temperatures through shading and evapotranspiration^4,5^ and help mitigating extreme heat^6^. Comparatively less is known, however, on the cooling potential of urban blue infrastructure elements, i.e. urban blue spaces^7^. Generally, cooling effects of water and vegetation follow different physical principles. Vegetation of green infrastructure elements perform by solar radiation obstruction and/or humidity increase^8^ and are effective in cooling particularly through shading for canopied sites^6^. In contrast, due to the high heat capacity of water, blue spaces generally heat up and cool down more slowly than land area, resulting in surface temperature differences that can cause cooling by local ventilation, known on a large scale as sea breeze systems^9,10^. Evaporative cooling also contributes significantly to the regulation of both surface and air temperatures by absorbing heat during the evaporation of water^11^. The difference in cooling mechanisms between vegetation and water bodies leads to distinct effects on actual cooling potential, including meteorological conditions as well as human thermal perception^12^.

The cooling effectiveness of blue spaces - such as lakes and ponds – depends on additional factors including the time of the day^13^. During the day blue spaces are very effective in mitigating heat through evaporative cooling and absorption of radiance^11,14^. However, studies have shown that blue spaces can also contribute to heating effects, as they release stored heat when water temperature exceeds air temperature. This heating effect is more pronounced during calm summer nights. During these periods, minimal air movement causes the heat stored in the water to be slowly released into the surrounding area. As there is barely any wind to disperse the heat, the warm and humid air remains above the water, causing nighttime temperatures in the local surrounding to rise^15^.

The cooling effect of blue spaces can also vary in different seasons with strongest cooling effects observed in spring and summer^7,11,16^. The stronger cooling during warmer months can be attributed to increased surface evaporation caused by increased solar radiation and higher air and surface temperatures in general^17^.

Land use types surrounding blue spaces significantly influence surface and air temperatures differently^18^, which in turn can influence the thermal effect of blue spaces on the local environment. Different land use types show different specific heat storage capacities. Land use types with a high proportion of impervious or mineral building material contribute to increased heat storage and the urban heat island in urban areas^19^. In contrast, land uses with a high proportion of vegetation show a lower heat storage capacity, and also provide cooling through evapotranspiration and shading. Green spaces surrounding urban blue spaces, thus, contribute to an enhanced cooling effect of blue spaces^20^.

Evaluating the cooling or even heating effects related to blue spaces characteristics requires precise metrics. The parameters of surface temperature cooling intensity and distance are metrics that have been used to quantify the overall cooling effect of blue spaces in various studies. These metrics describe the continuous surface temperature increase over a certain distance. While the location where the continuous increase in temperature occurs defines the cooling distance, the temperature difference between the water body and the specific location describes the intensity^16,17,22^. Additionally, the Threshold Value of Efficiency (TVoE) suggests a cooling efficiency from a cost-benefit perspective and may serve as a parameter for urban planners when designing cities adapted to mitigate heat^24^. The TVoE relates the surface temperature cooling intensity or distance to the area of the blue space and is context dependent to additional parameters such as local climate conditions, surrounding land use, urban morphology, and wind exposure^23^. The TVoE indicates the size at which the water body achieves maximum surface temperature cooling effectiveness relative to its very specific area^20,21,26^.

The area or size of a blue space is, thus, an important factor in a blue space’s thermal effect on the local urban environment. Recent studies consistently show that larger blue spaces have a greater cooling effect, especially in the warmer seasons of spring and summer^7,27^. The greater cooling effect of larger blue spaces can be attributed to the greater thermal mass, which allows them to absorb and store more short-wave solar radiation and keep surface temperatures stable through thermal mixing^28^. As a result, larger blue spaces have less significant temperature fluctuations and a more uniform cooling effect. In contrast, smaller blue spaces tend to heat up faster during hot periods due to their limited volume, resulting in higher water surface temperatures and heating effects^20,28,29^.

To conclude, a number of factors such as the size of blue spaces, season and specific land use types contribute to the thermal impact of blue spaces on the local environment. Research and related interpretation on the factors’ combined contribution is, however, limited by the selection of assessment method. In-situ based environmental measurements are able to assess such multiple factors to address thermal comfort such as air temperature, wind, air humidity rather than surface temperature, and may more precisely reflect the cooling potential of blue spaces in regard to human thermal comfort. These in-situ based assessments face, however, challenges such as (i) a lack of standardized protocols e.g. for measuring air temperature limiting cross-study comparisons; (ii) difficulties in converting in-situ based point or transect observations into spatially explicit maps allowing assessments at various locations^6^; and (iii) the requirement of resources (budget, time, staff) limiting spatial and temporal coverage^30^. Remote sensing-based assessments, on the other side, use surface radiation and derived surface temperatures over larger areas but are not able to provide direct air temperature, wind and air humidity values needed to fully assess human thermal comfort^6^. Remote-sensing based approaches, however, would allow us researching trait variations of objects – such as surface temperature of blue spaces and their local environmental conditions including local landuse structure types – over space and time on a larger scale such as for an entire city^31,32^ and can follow standardised protocols and methods.

The aim of this study is, thus, to assess the surface thermal effect of urban blue spaces on the local environment during different seasons of the year and considering different blue space characteristics such as size of blue spaces and surrounding land use types applying a remote-sensing based assessment. We ask:

1. How does the surface thermal effect of blue spaces vary across different seasons and with different lake sizes?
2. What is the *Threshold Value of Efficiency* in relation to the size of the lakes?
3. How is the surface temperature of blues spaces related to surrounding urban land use types and urban morphological factors?

Using the city of Hannover, Germany, as a case, we apply a Geoinformation System (GIS) approach, use remote sensing-based images and landuse and landcover information to assess the surface thermal effect of different lake sizes of the city.

## Materials and methods

### Study area

**Fig. 1:**
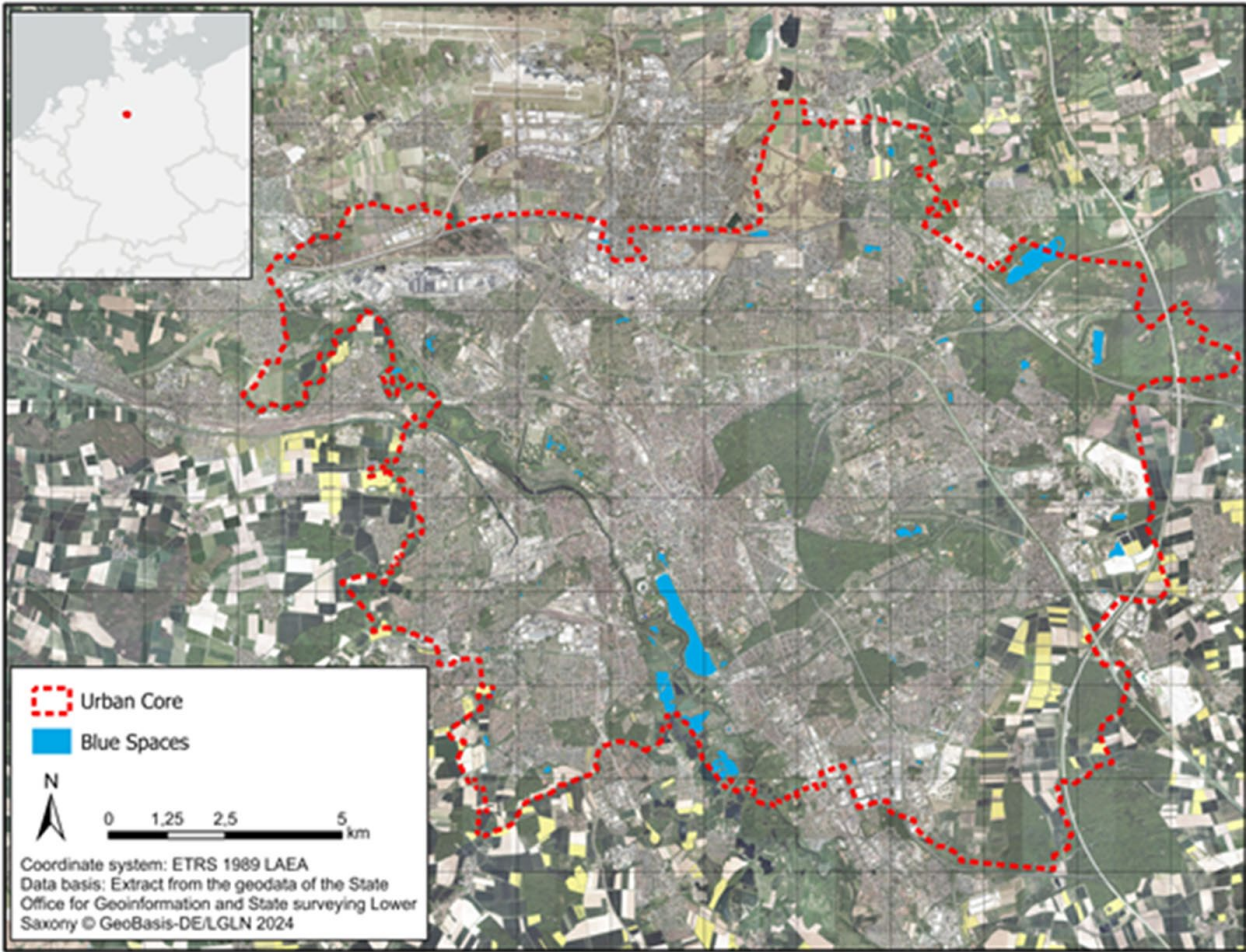
Hannover case study with analysed blue spaces inside the administrative city boundary. Note: Map was created by authors with ArcGISPro software (ESRI, Version 3.1.1) based on open source geodata provided by the LGLN (State Office for Geoinformation and State Surveying Lower Saxony, 2024, https://ni-lgln-opengeodata.hub.arcgis.com/apps/lgln-opengeodata::digitales-orthophoto-dop/about^38^.

The city of Hannover (52.3759° N, 9.7320° E) is the capital and largest city of the federal state Lower Saxony located in northern Germany (Fig. 1), with an estimated population of around 558,258 in 2025, making it the 13th largest city in Germany in terms of population^33^. Hannover falls within the Cfb climate zone under the Köppen-Geiger classification^34^. This classification identifies the city as a temperate, oceanic climate zone, characterized by mild summers, cool winters, and relatively even precipitation distribution throughout the year. The mean annual air temperature for the period 1991 to 2021 is 10.3 °C with variations between the warmest month in July (19.1 °C) and coldest month in January/February (−0.2 °C). The mean precipitation amount of 790 mm is evenly distributed throughout the year with highest amount in July (82 mm) and lowest in February (51 mm)^35^.

We choose Hannover as a case study, because Hannover is a large city with a diverse urban landscape, containing multiple land use type classes that reflect the city’s diverse infrastructure, green and blue spaces including blue spaces of very different sizes. Urban forests cover 10.6% of the city’s area, while green urban areas account for 9.2%. The biggest lake in the city - the Maschsee, an artificial lake of 77.5 ha located near the city centre, contributes to the 1.8% of the urban area covered by water^36^. Due to the high amount of urban land use classes characterized by a high share of impervious areas in the inner-city area (55.8%), Hannover shows characteristics of the urban heat island with elevated temperatures in the urban city centre compared to its surrounding rural areas. The combination of extreme heat and the local urban heat island effect has led to a significant increase in the number of heat days and tropical nights for the central inner city area of Hannover illustrating the high importance of implementing effective heat mitigation strategies^37^.

### Data

#### Selection of relevant blue spaces

We used the Urban Atlas Land cover data^36^ for the identification and selection of the blue spaces aiming to include a wide range of lakes of different sizes and surrounding land uses guided by three criteria: First, blue spaces larger than 0.09 ha were chosen, corresponding to data resolution of the Land Surface Temperature (LST) data (see below), which ensures compatibility and precision in thermal data acquisition. Second, a minimum distance of 90 m should be maintained between selected blue spaces to exclude an overlapping thermal effect of other blue spaces and to ensure clear thermal signatures. Third, we considered variation in size to capture a diverse range of blue spaces and possible variation in thermal effects in relation to lake size. The selection criteria follow approaches of earlier studies^16,39^. The selection analysis resulted in a number of 79 blue spaces covering areas between 0.1 ha to 77.5 ha, respectively.

To determine the surface thermal cooling intensity and distance of these blue spaces, a buffer analysis in a 50-meter interval up to 1000 m from the lake boundaries was performed. We used the buffer distance of 50 m because this distance is greater than the resampled 30-meter resolution of the Land Surface Temperature (LST) data and results in a more robust data basis^40 ^while a small-scale analysis is maintained by the interval of the buffer zones, enhancing the reliability of our analysis. All spatial analyses and visualisations were done with ArcGIS Pro 3.1.1 by ESRI.

#### Land surface temperature (LST)

Remote sensing data obtained from the Earth Explorer platform provided by the United States Geological Survey^41^ were used for LST identification. We focused on Landsat 8–9 Operational Land Imager (OLI) and Thermal Infrared Sensor (TIRS) Collection 2 Level 1 imagery. To ensure data quality and reliability, only images with cloud cover less than 10% were selected. The temporal scope of the study encompasses all four seasons and multiple years for the analysis to be more representative of the seasonal results compared to only using a single year (Table 1). Though efforts were made to obtain comparable observations for each season, this was only possible to a limited extent due to data availability caused by cloud cover and the limited temporal resolution of the Landsat mission (approximately one image every 16 days), which restricted the number of suitable images. Multiple years from December 2021 to October 2024 allow a comparison between different years and seasons. We selected data from the years 2021 to 2024 because their seasonal mean temperatures represent an average summer situation for Hannover compared to the long-term mean^42^. In contrast, for instance the years 2018 to 2020 were characterised by extreme heat throughout Germany, including Hannover^37^. By focusing on the more typical climate period from 2021 to 2024, the study aims to provide insights that are more generally applicable to understanding seasonal and annual climate variability in this region. In this study, the winter season is defined as the period from December of the previous year to February of the current year, i.e. winter 2022 begins in December 2021 and lasts until February 2022. The remote sensing data were processed into Land Surface Temperature data using the Semi-Automatic Classification Plugin (SCP) for QGIS^43^.

**Table 1:** Overview of the images used for the analysis with min., max. and mean LST temperatures for the city of Hannover.

Season	Date	Local time	Min/Max/Mean LST in °C
Winter	20.12.2021	11:15	−5.6/6.0/0.3
	15.12.2022	11:15	−8.0/3.8/−3.0
	27.01.2024	11:15	−7.1/13.1/3.3
Spring	18.04.2022	12:20	11.3/37.5/20.0
	30.04.2023	12:15	11.4/37.3/18.6
	01.05.2024	12:20	15.5/38.4/23.8
Summer	24.07.2022	12:15	21.5/44.1/30.4
	11.07.2023	12:15	12.2/42.3/28.1
	13.08.2024	12:20	21.8/38.8/27.4
Autumn	02.09.2022	12:15	6.4/35.3/24.6
	05.09.2023	12:15	20.4/40.7/26.7
	25.10.2024	12:15	7.1/22.2/14.1

Note: All images are a product by Landsat 8–9 OLI/TIRS C2 L1 with Band 10 and a native thermal resolution of 100 m but are provided as surface temperature data on a 30 m grid after interpolation/resampling.

To qualitatively discuss a potential influence of the background climate condition on surface thermal effects of blue spaces, meteorological data from the closest German Weather Service (DWD) station for Hannover (the only one available with DWD), located at the border of the city were used (see supplementary material). Data for air temperature, relative humidity, and wind speed were used to describe the general context weather situation, while acknowledging that the very local urban conditions may deviate from the station measurements. No further quantitative assessments could be done since data is limited to one station.

### Data analysis

**Fig. 2:**
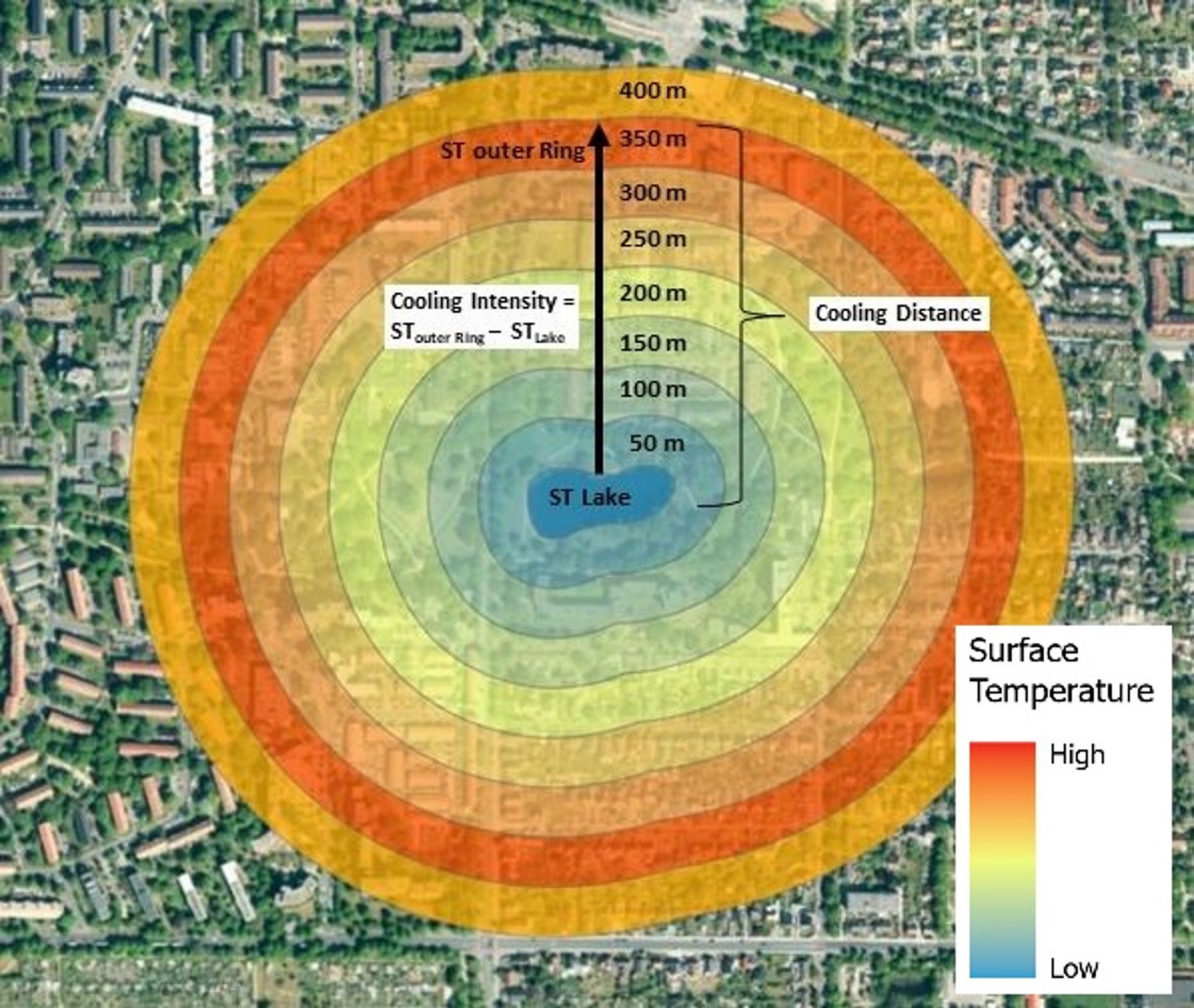
Scheme for determining the surface cooling distance and cooling intensity based on the Mean Surface Temperature within the individual buffer rings. The figure illustrates, as an example, the method for quantifying surface cooling effects based on mean surface temperature within concentric buffer rings. Buffers are shown as colored zones extending from the water body (blue, cooler) to 400 m distance (orange, warmer) over an aerial image of Hannover. Each buffer ring represents the mean land surface temperature (LST) of that zone. Since the outermost ring at 400 m shows a slightly lower mean LST than the 350 m ring, the cooling distance is determined as 350 m—the point at which the mean temperature no longer consistently increases with distance from the water. Arrows and annotations indicate the determination of cooling distance and cooling intensity, calculated as the difference between the water surface temperature and the outermost relevant buffer ring.

To calculate the surface thermal effect of urban blue spaces with intensities and distances, zonal statistics were applied to the respective LST datasets, utilizing the outlines and buffer rings of the blue spaces, as detailed in Chap. 2.2. The surface temperature cooling or heating distance is calculated by examining the number of buffer zones at 50-meter intervals following an established approach by earlier studies^21,44^ over which the mean temperature consistently increases or decreases in relation to the previous buffer zone. The point (spatial distance point) at which these increases or decreases in temperature end, indicates the surface temperature cooling or heating distance^21^. The surface cooling or heating intensity is then derived from the mean surface temperature difference between this outermost buffer ring and the water surface temperature of the blue space itself (Fig. 2). The distributions of water surface temperature, cooling/heating intensity, and cooling/heating distance (CD) are presented using boxplots. Outliers for WST, CI, and CD were identified using the 1.5 × interquartile range (IQR) criterion.

Based on size and following a study by Wang and Ouyang 2021^27^, the calculation of the Threshold Value of Efficiency (TVoE) was calculated in consideration of the efficiency of each blue space inside the urban boundary. In this case, the TVoE describes the most efficient lake size for urban surface cooling, meaning that lakes larger than the TVoE may show stronger but less efficient surface temperature reduction effects in relation to their area. The surface cooling efficiency is defined by considering the ratio between the lake’s size and its absolute water surface temperature, cooling intensity and distance. To calculate the TVoE, the parameters of water surface temperature, surface temperature cooling intensity and distance can be set in context with its patch area. The TVoE for all blue spaces are represented as a logarithmic curve, plotting the values of the parameter against lake size. The point on this curve, where the value of the parameter begins to stagnate with increasing lake size, is, then, defined as the Threshold Value of Efficiency^25,27^.

#### Urban land use types surrounding blue spaces and morphological factors

**Fig. 3:**
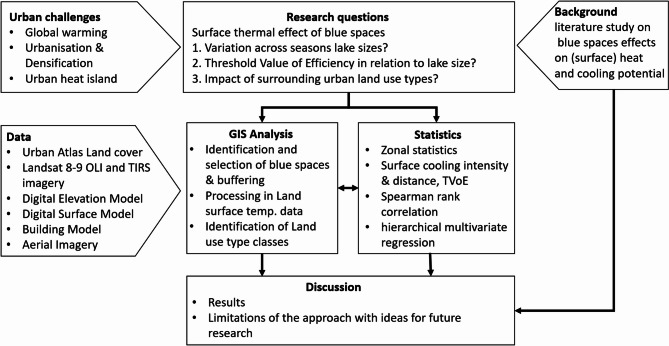
Schematic illustration of the overall methodological workflow, showing the consecutive steps from data acquisition to analysis.

Various geospatial datasets were combined and analysed in the first buffer zone (50 m) around each blue space, as the immediate surroundings (50 m) have been shown to significantly influence water temperatures^50^: (1) building model, (2) aerial imagery with 20 cm resolution (3) digital elevation model (DEM), (4) digital surface model (DSM) both with 1 m resolution and (5) Urban Atlas Dataset (Fig. 3).

The RGBI aerial imagery was processed to derive the Normalized Difference Vegetation Index (NDVI). A structural height raster (nDSM) was created by subtracting the digital elevation model (DEM) from the digital surface model (DSM). High vegetation as tree locations were then defined by linking the structural height (> 3 m) with the vegetation cover defined from the NDVI. The threshold value of 3 m was chosen to separate trees from lower vegetation, consistent with the FAO classification, which allows woody vegetation taller than 3 m to be classified as trees when a distinct tree-like physiognomy and canopy structure is present^45^. Blue spaces were identified from the Urban Atlas dataset and, like the building model, rasterized at a 1-meter cell size. Finally, all data sets were combined to create a 1-m resolution grid containing the land use type classes most relevant for LST analysis due to their thermal characteristics^18^: (1) Buildings, expressed as Building Cover Ratio (BCR), (2) Impervious areas and bare soil, (3) Low Vegetation, (4) High Vegetation and (5) Blue Spaces. Based on this land use raster, the percentage share of each land use class (as continuous variable) was conducted for a 50-meter buffer around each blue space.

In addition to land use type information, selected urban morphological/geometry parameters were used. The Sky View Factor (SVF) was chosen because it quantifies the degree of sky openness and shading, thereby influencing radiative cooling and heat storage. Distance to built-up areas was considered to account for the broader urban context. The SVF was calculated with SAGA GIS^46^ using a combined elevation model, where vegetation heights were taken from the nDSM and building heights taken from a 1-meter building height grid generated from the building model. The mean building height was calculated from the same building height grid. The values for both parameters were extracted for each 50-meter buffer, as was the land use types share.

Distance to built-up areas was determined to describe the spatial relationship between blue spaces and the distance to the surrounding built environment. Concentric 10-meter rings were created around each blue space, and the building coverage was calculated for each ring. Distance to built-up areas was defined as the distance to the first ring in which the building coverage exceeded 10%, representing the transition to a predominantly built-up area. The threshold value of 10% was chosen because it corresponds to the lower limit of built-up areas as defined in the Local Climate Zone classification^47^. We carried out a Spearman’s rank correlation analysis to identify a potential relationship between the area coverage ratio of the land use classes in the surrounding of the lakes (50 m buffer) and their water surface temperature, cooling intensity and cooling distance.

To further assess the relative importance of structural drivers, we applied a hierarchical multivariate regression analyses separately for each year and season. In the models, we treated landuse structure variables as confounders and the urban morphological variables and an interaction between SVF and building height as predictors to predict surface cooling intensity (CI) and surface cooling distance (CD). All predictor variables were converted to standardized Z-scores prior to model estimation to ensure comparability between variables measured on different scales^48^.

Figure 3: Schematic illustration of the overall methodological workflow, showing the consecutive steps from data acquisition to analysis. 

## Results

### Surface thermal effect of blue spaces in different seasons

Surface temperature cooling or heating distance is defined as the spatial extent from the blue space shoreline over which mean land surface temperature in successive 50-m buffer rings consistently increases or decreases relative to the preceding ring. Surface cooling or heating intensity represents the surface temperature difference between the outermost buffer ring where this trend ends and the lake surface temperature.

#### Water surface temperature

**Fig. 4:**
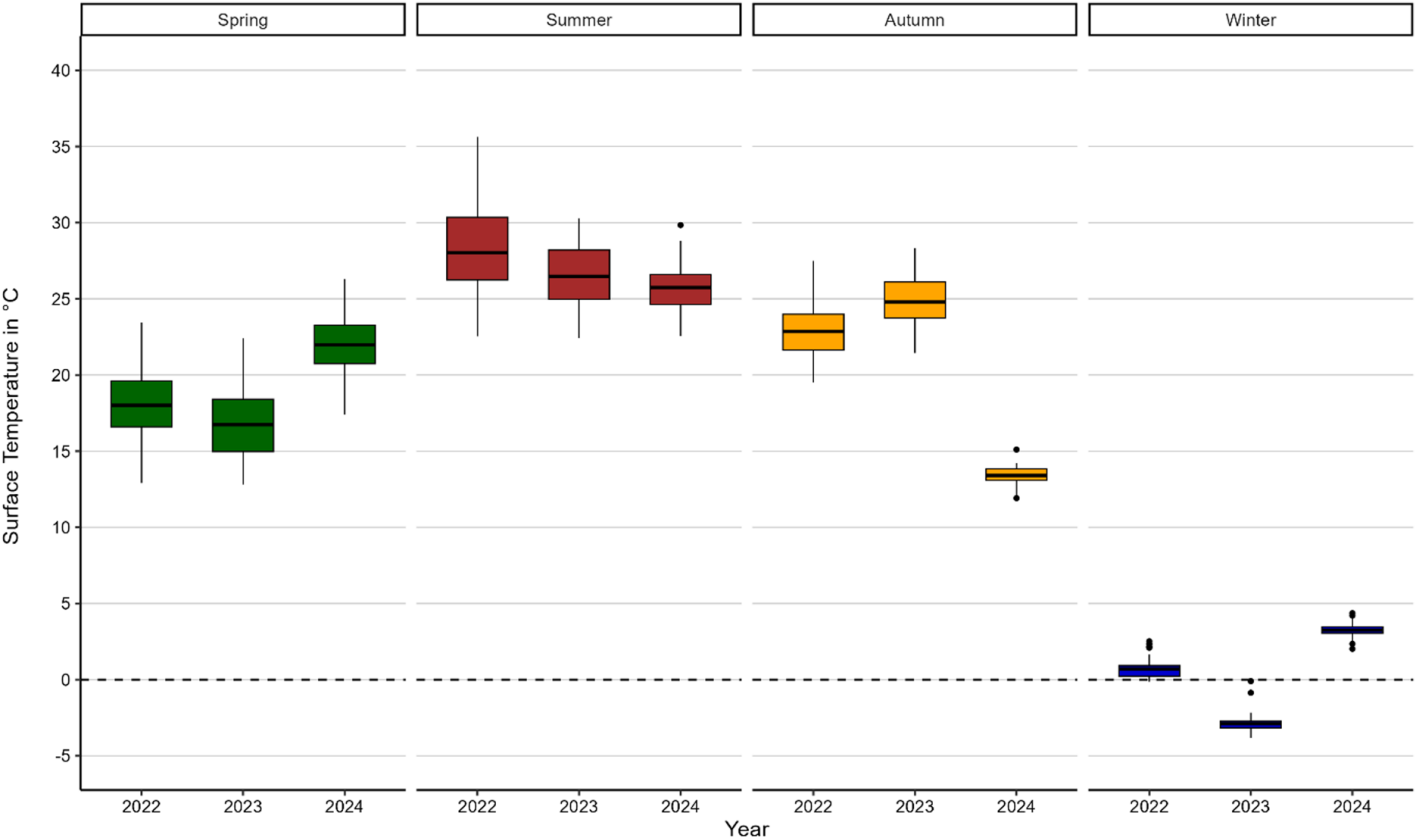
Comparison of water surface temperature variations of lakes in different seasons and years. Boxplots show the water surface temperature of the analysed lakes for four seasons: spring, summer, autumn, and winter (from left to right). Within each season, data from three years (2022–2024) are presented, illustrating seasonal patterns and year-to-year variability in lake surface temperatures.

Figure 4 shows that the water surface temperature (WST) of lakes in Hannover varies significantly across seasons depending on the years of analysis. Generally, the highest seasonal averaged surface temperatures of the lakes are recorded in summer (26.7 °C), followed by autumn (20.4 °C), spring (18.9 °C) and winter (0.4 °C). Across all 3 years, spring season shows the highest average range (9.7 °C), followed closely by summer (9.4 °C), autumn (6.0 °C) and winter (2.9 °C).

We found significant interannual differences with notably lower surface temperatures in autumn 2024, which averaged at 13.4 °C compared to the warmer autumns of previous years (24.8 °C in 2023 and 22.9 °C in 2022). The comparatively lower values for the winter in 2023, with an average surface temperature of −2.9 °C differs to the comparatively milder winters of 2022 (+ 0.7 °C) and 2024 (+ 3.2 °C).

Highest outlier observed for summer 2024 can be attributed to a very small lake (0.12 ha; 29.8 °C). In autumn 2024, the identical small lake accounted again for an outlier showing comparatively warmer surface temperatures (15.1 °C).

#### Surface temperature cooling and heating intensity

**Fig. 5:**
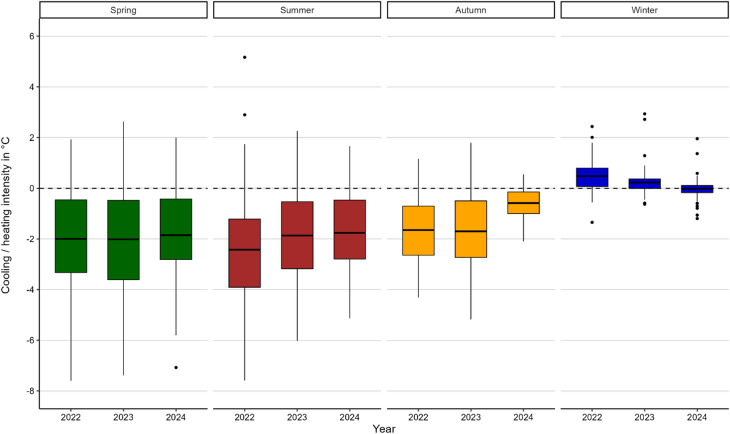
Comparison of variations in heating and cooling intensities of lakes in different seasons and years. Positive values represent heating intensities, negative values cooling intensities. Boxplots show the surface cooling intensity of the analysed lakes for four seasons: spring, summer, autumn, and winter (from left to right). Within each season, data from three years (2022–2024) are presented. Negative values indicate cooling effects, whereas positive values represent heating effects, highlighting seasonal and interannual variability in the thermal influence of the blue spaces.

Surface temperature cooling intensities are identified across all seasons and years (as indicated by values below zero in Fig. 5). Average surface cooling intensity for all years is shown to be highest in summer and spring (both − 2.0 °C). Autumn data show a moderate lower intensity with an average of −1.3 °C, while winter shows a unique pattern with an average that is slightly positive at 0.2 °C, meaning that surface thermal effects of heating slightly dominate for this season.

The maximum surface thermal effect with cooling intensities averaged over all years are highest in spring (7.4 °C), followed by summer (6.2 °C), autumn (3.9 °C), and winter (1.1 °C). However, averaged maximum surface heating intensities are greatest in summer (3.0 °C). The range between maximum surface cooling intensity and minimum surface cooling respectively maximum heating intensity over all 3 years is largest in spring (9.5 K) and summer (9.3 K) and smallest during winter (3.5 K).

Summer 2022 showed two high outliers, both associated with very small lakes with a size of 0.10 and 0.13 ha respectively and high surface temperature heating intensities (2.9 °C and 5.2 °C). In winter 2022, another small lake of 0.2 ha showed highest cooling intensity (1.3 °C).

#### Surface cooling and heating distance

**Fig. 6:**
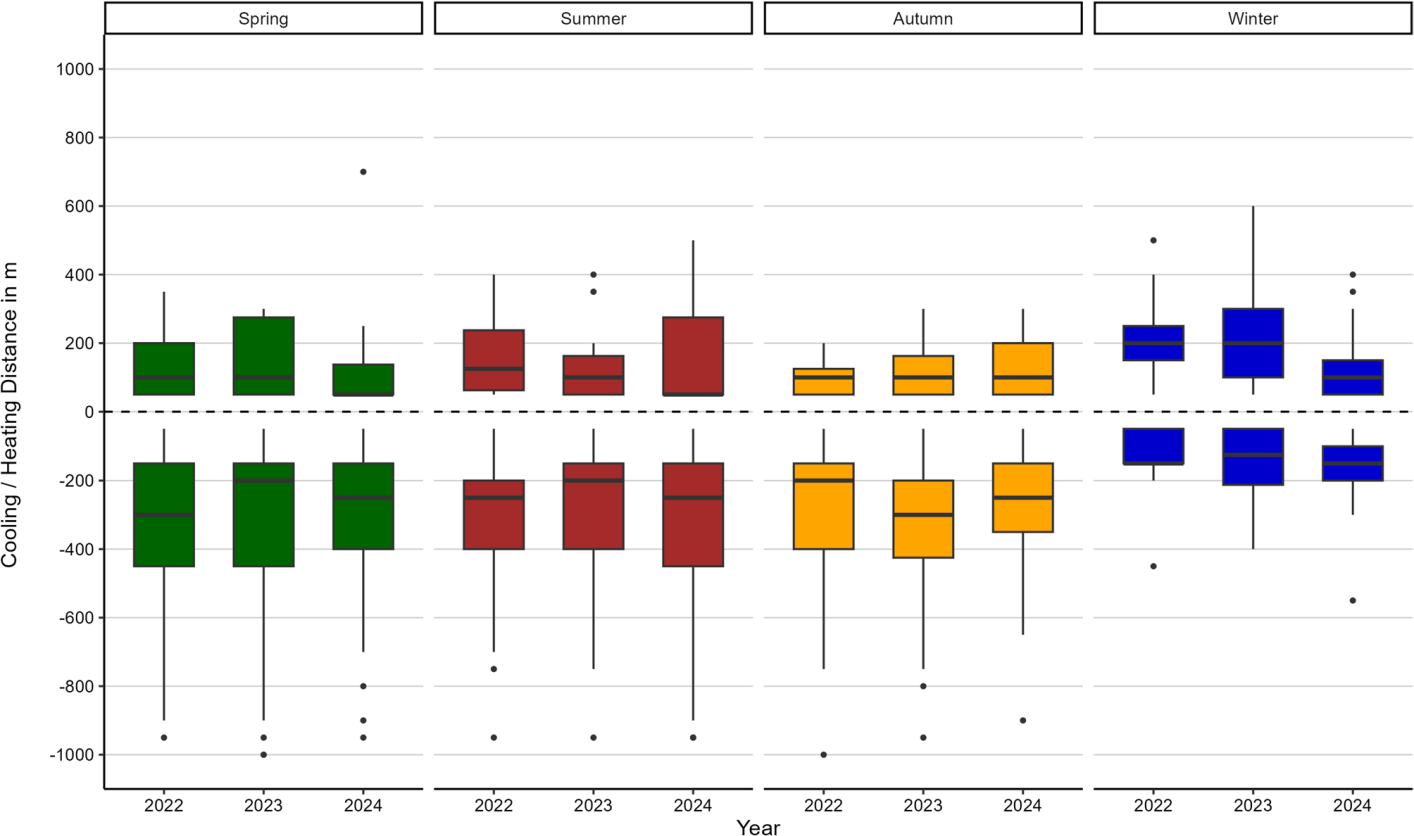
Comparison of heating and cooling distances of lakes in different seasons and years. Positive values represent heating distances, negative values cooling distances. Boxplots show the surface cooling distance of the analysed lakes for four seasons: spring, summer, autumn, and winter (from left to right). Within each season, data from three years (2022–2024) are presented, illustrating seasonal patterns and year-to-year variability in the spatial extent of the cooling effects of the blue spaces.

Overall, the highest mean surface thermal effect relating to distances averaged over all years could be identified for lower surface temperatures in summer (311.6 m), and spring (308.5 m) (Fig. 6). However, in winter, the averaged surface cooling distance occurred at a notably lower level (146.3 m). In contrast, mean surface heating distances are highest for winter (182.3 m), and lowest for autumn with 114.5 m.

Comparing averaged and maximum surface temperature cooling and heating distances, cooling distances are on a significantly higher level than the heating distances in spring, summer and autumn, meaning that during these seasons, the surface temperature cooling effects emanating from the lakes are significantly stronger than potential surface temperature heating effects. In contrast, winter values show higher averaged and maximum heating distances than cooling distances meaning that cooling distances are more spread during spring, summer and autumn compared to heating distances, while the range is similar during winter.

Outliers in heating distance with higher values indicate that across all seasons and years, a total of three small lakes with an area of 0.2 to 0.3 ha show high cooling distances. These lakes are centrally located in very large green spaces, which could contribute to their thermal effect.

### Thermal effect of blue spaces of different sizes

**Fig. 7:**
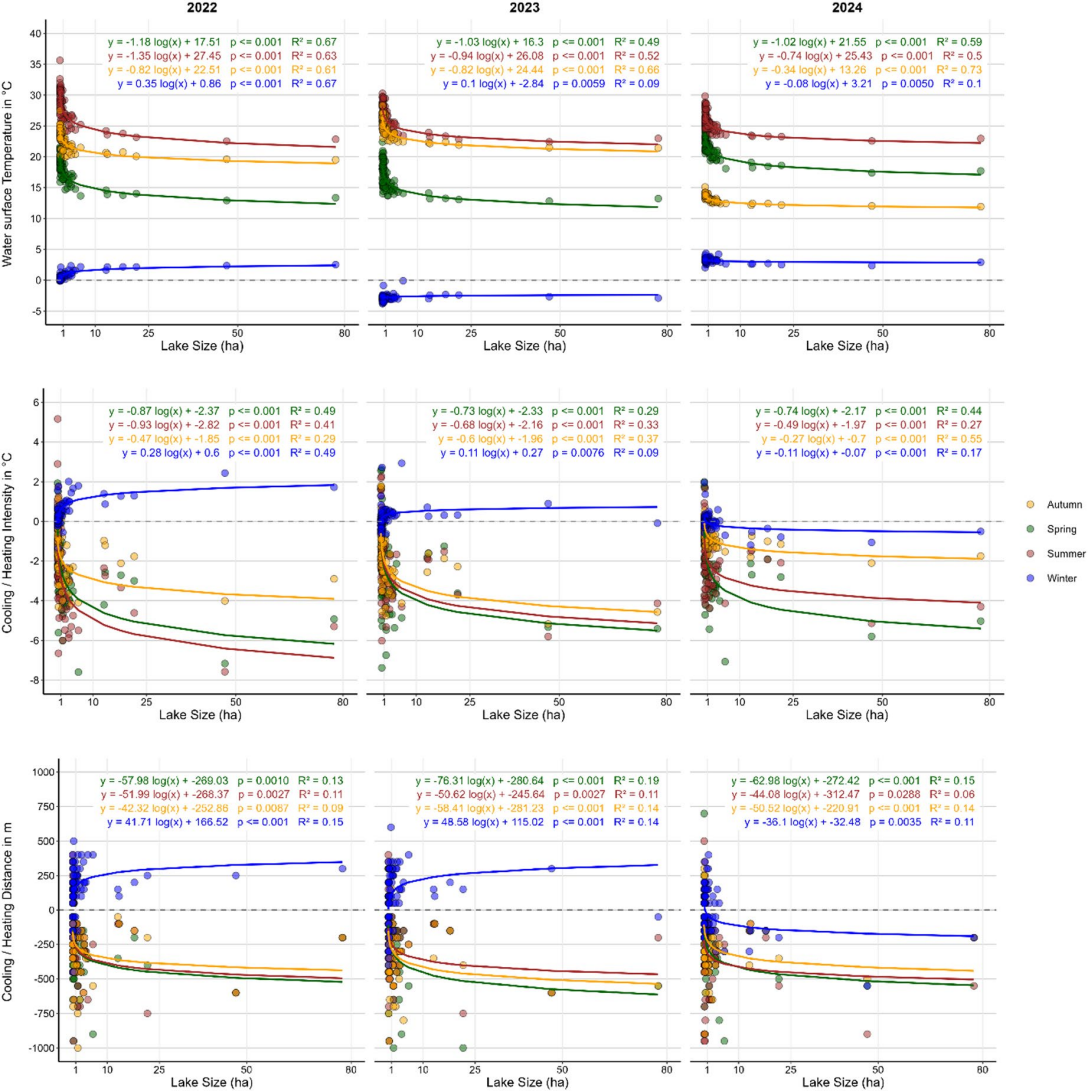
Logarithmical relationship between water surface temperature (top), surface temperature intensity (middle) and Distance (bottom) of blue spaces and the lake area. The panels illustrate the relationship between lake size and water surface temperature (top), surface cooling intensity (middle), and cooling distance (bottom). Based on these logarithmic relationships, the Threshold Value of Efficiency (TVoE) was derived, indicating the lake size most effective for achieving cool water surface temperatures, high cooling intensity, or extended cooling distance. Each plot represents a single year (2022 left, 2023 middle, 2024 right) and includes data from all seasons, shown in distinct colors to highlight seasonal variability.

In Fig. 7, the indicators for analysing the thermal effect of blue spaces (WST, CI and CD) are plotted in relation to their specific lake area size to identify the TVoE. During summer, the TVoE for a cool surface water temperature varies between 1.35 ha in 2022 and 0.74 ha in 2024 with an average value for all years analysed of 1.01 ha, meaning that lakes smaller 1.01 ha are on average warmer in their surface temperature than larger ones and lakes larger 1.01 ha do not show significant additional decreases in WST in relation to its increasing patch size. The logarithmic relationships between lake size and WST are highly significant during spring, summer, and autumn (*p* < 0.001) and exhibit strong explanatory power (R² up to 0.74), whereas this relationship is much less significant for winter values, as indicated by markedly lower R² values and reduced statistical significance.

We found that for surface cooling intensity a TVoE of 0.70 ha (averaged for all years analysed) suggests that lakes of this particular size are effective in cooling surfaces of the local urban environment during the hottest months in summer, without significant gains in values of cooling for lakes with larger areas. Highest TVoE for all seasons occurs in Spring (0.78 ha), but remains at a comparable level to summer. While lowest TvoE occur in autumn (0.27 ha), results in winter show, as for WST, that larger lakes have greater heating effects. The relationships between lake size and surface cooling intensity are statistically significant in most seasons (*p* < 0.001) with moderate explanatory power (R² ≈ 0.27–0.55), confirming a size-dependent cooling response, while weaker relationships in winter indicate a reduced influence of lake size under cold-season conditions.

In summer, the TVoE for surface cooling distance is on average 48.90 ha, which indicates that larger lakes distribute their cooling impact more effectively over greater distances. Greater TVoE occur both in autumn (50.42 ha) and spring (65.76 ha), meaning that larger lakes show higher cooling distances in these seasons. The logarithmic relationships between lake size and surface cooling distance are statistically significant (*p* < 0.01) in most seasons, but have low explanatory power (R² ≈ 0.06–0.19). In summer, the relationship is weaker and less consistent, suggesting that the spatial extent of cooling is more strongly influenced by additional factors than by lake size alone.

### Impact of surrounding land use types and urban morphological characteristics on blue spaces’ surface thermal effect on the urban microclimate

**Fig. 8:**
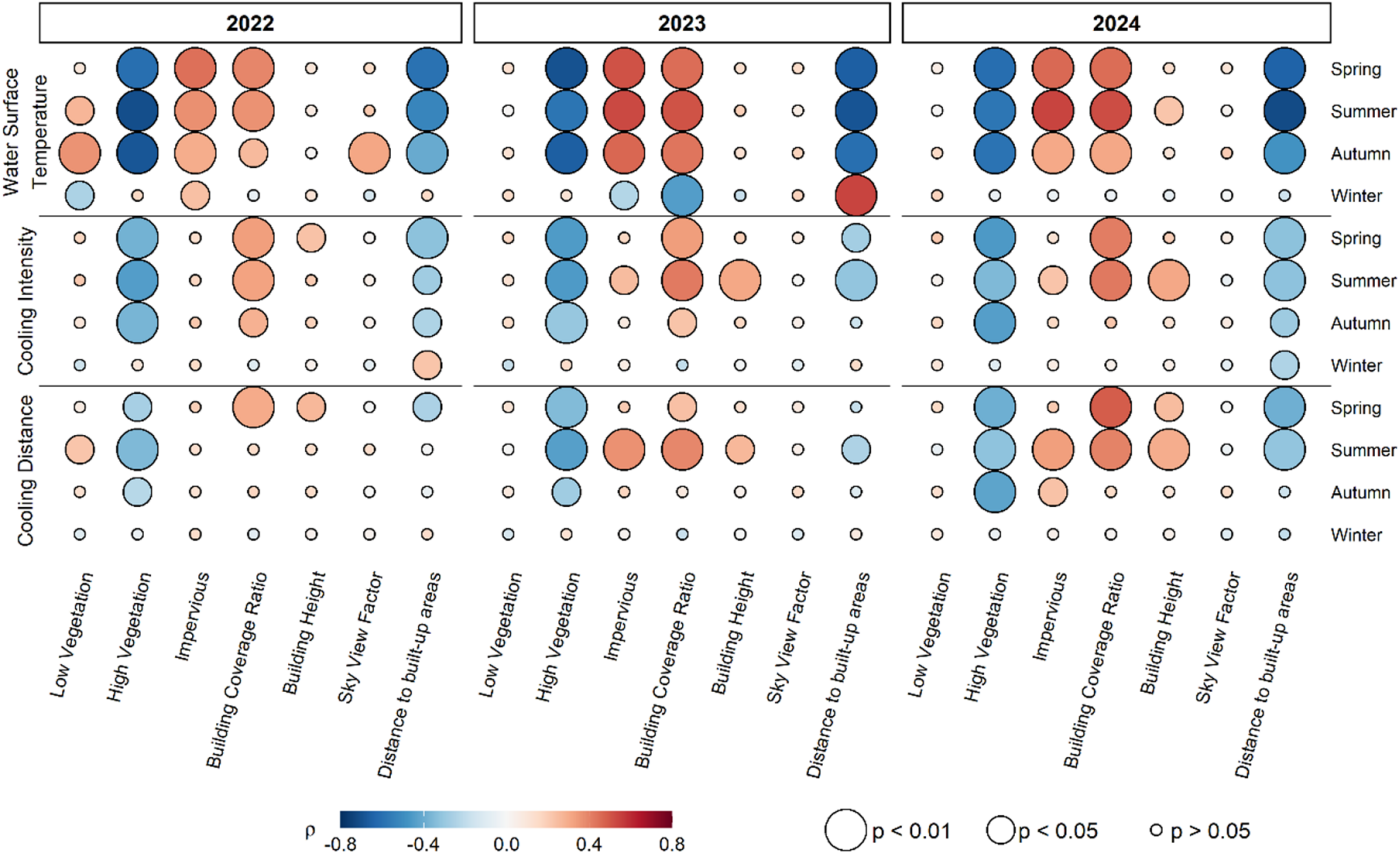
Association between land use classes and urban geometric parameters and the parameters water surface temperature, surface cooling intensity and distance. The top panel show bubble heat maps representing Spearman’s rank correlations between land use classes (Low Vegetation, High Vegetation, Building Cover Ratio, Impervious areas) and the parameter of water surface temperature (WST). Bubble color indicates the correlation strength (dark blue: strong negative; white: no correlation; dark red: strong positive), while bubble size reflects statistical significance (large: *p* < 0.01; medium: *p* < 0.05; small: *p* > 0.05). WST correlations are displayed in the upper section, arranged in four rows for spring, summer, autumn, and winter. Years are presented from left to right (2022, 2023, 2024). Panels below WST show corresponding correlations for surface cooling intensity and cooling distance, following the same layout and color/size coding.

The surface temperature of the lakes analysed shows significant associations with various land use type classes and urban geometric parameters, specifically “Impervious areas and bare soil”, “High Vegetation” such as trees, “Building Cover Ratio”, and “Distance to built-up areas” (Fig. 8). For example, during summer 2022, a strong negative association was observed between the “High Vegetation” class and WST (ρ = −0.709, *p* < 0.01), indicating that a higher presence of trees is associated with a decrease in water surface temperature. In contrast, the presence of buildings in the same season was positively related to WST (ρ = 0.363, *p* < 0.01), suggesting an increase in temperature. These patterns are consistent across subsequent years and apparent across most seasons, except for winter, where no significant correlations were detected. Distance from urban areas showed the most consistent associations among all parameters analysed. Greater distance from urbanized areas was significantly associated with lower WST values (e.g., summer 2024: ρ = −0.719, *p* < 0.01; spring 2024: ρ = −0.648, *p* < 0.01), suggesting that lakes further away from densely built-up areas tend to remain cooler. Average building height did not show a consistent pattern across years or seasons. Also, SVF did not show a consistent association with WST. Correlation coefficients were generally weak and non-significant across most seasons and years. Only for autumn 2022 a moderate positive association was observed (ρ = 0.312, *p* < 0.01), indicating slightly higher surface temperatures under more open sky conditions.

The lakes’ surface cooling intensity (CI) shows significant associations with the land use classes and urban geometric parameters, particularly “High Vegetation”, " Building Cover Ratio " and “Distance to built-up areas”. For example, in the summer of 2023, a significant negative correlation was identified between High Vegetation type and CI (ρ = −0.456, *p* < 0.01), which means that a high share of high vegetation in the surroundings of blue spaces leads to increased cooling intensities. Building Cover Ratio, in contrast, shows a positive correlation in summer 2023 (ρ = 0.419, *p* < 0.01), meaning that an increased share of buildings in the vicinity of blue spaces leads to a decreased surface temperature cooling and increased heating intensity. These patterns were identified for all years and seasons analysed, with the strongest negative correlations between CI and high vegetation in spring 2024 (ρ = −0.459, *p* < 0.01). For the winter season, no significant correlations were observed. Distance to built-up areas showed similarly consistent effects on CI. In all non-winter seasons, significant negative correlations were detected (e.g., summer 2024: ρ = −0.326, *p* < 0.01; spring 2024: ρ = −0.328, *p* < 0.01), indicating that lakes situated more distant from urbanized surroundings tend to generate stronger surface cooling intensities. Mean building height exhibited significant positive correlations with CI mainly during summer and occasionally in spring (e.g., summer 2023: ρ = 0.311, *p* < 0.01), suggesting that taller surrounding buildings reduce cooling intensity. In contrast, SVF showed no significant association with CI in any season or year. This indicates that sky openness does not substantially influence the magnitude of surface cooling intensity compared to land cover composition and urban structure.

Surface cooling distance (CD) shows significant associations with the land use classes “High Vegetation” and " Building Cover Ratio” and “Distance to built-up areas”. During the summer of 2023, a notable negative correlation was found between the presence of trees and cooling distance (ρ = −0.441, *p* < 0.01), suggesting that a higher proportion of trees around blue spaces leads to longer cooling or smaller heating distances respectively. Conversely, as for CI, the presence of buildings during summer shows a positive association with cooling distance (ρ = 0.388, *p* < 0.01 in 2023), implying that a high share of buildings increases the range of heating effects or decreases the range of cooling effects respectively. In subsequent years, a similar pattern emerges, with some of the most pronounced negative correlations for trees occurring in autumn 2024 (ρ = −0.426, *p* < 0.01). Again, these trends are apparent in every season but winter. “Distance to built-up areas” is also significantly related to CD, particularly in summer seasons (e.g., 2024: ρ = −0.315, *p* < 0.01), indicating that lakes located more distant from urban areas tend to influence larger surrounding areas. Mean building height showed only occasional significant positive associations with CD, primarily in summer and in some spring periods (e.g., summer 2024: ρ = 0.295, *p* < 0.01). Again, SVF did not show a statistically significant relationship with cooling distance across seasons and years indicating that the indicator of the SVF does not significantly contribute to explaining the spatial extent of lake-induced surface cooling.

To assess the relative importance of the indicators related to lake size, land cover, and urban morphology, hierarchical multivariate regression models were applied (see Supplementary Tables 2–9). Across all years and seasons, lake area and land cover structure variables consistently explained the highest share of variance, particularly for cooling intensity (CI), where model performance was highest in spring and summer (model 1 up to 40%). The inclusion of urban morphological variables (SVF, building height, urban distance and the interaction effect) as predictor variables resulted in a moderate additional explanation of variance. For cooling distance (CD), explanatory power was overall low, indicating that its spatial extent is less directly controlled by the investigated variables.

## Discussion

This study provides an analysis of the surface thermal effect of 79 lakes on the urban local environment in the city of Hanover across seasons and for three consecutive years. The selected lakes represent a broad size range from 0.1 to 77.5 ha, including many small water bodies that are, so far, less analysed in remote sensing-based studies on the topic^7^. Our results indicate that the surface thermal effect of blue spaces varies across different seasons and by water body size with highest surface cooling intensities for all lake sizes during summer and spring (both average value of 2.0 °C), with widest average cooling distances identified for the summer season in Hannover (311.6 m). The analysis of lake size showed for this study case that a Threshold Value of Efficiency of 0.70 ha is effective for surface cooling intensity and a TVoE of 48.90 ha for surface cooling distance. We identified significant negative association between different landcover structure types of buildings and impervious areas in the vicinity of blue spaces and surface cooling intensity and distance. Higher shares of high vegetation were significantly positively associated with lower surface temperature, higher surface cooling intensities, and distances. We, however, also showed that the identified influencing factors contribute only partly to the explanation of variance in the outcomes particularly in terms of cooling distance. We discuss further below, that more research is needed that consider additional impact factors such as the local wind regime, air humidity and water body depth as explaining variables.

As shown in other studies^7,20^ we identified that smaller lakes reflect a broader surface thermal range and greater sensitivity to seasonal temperature variations compared to larger lakes. Smaller lakes show mostly higher surface temperatures in summer and spring, and significantly lower minimum temperatures for the winter than larger lakes. These differences can be explained by the high heat storage capacity of water and the different volumes of the blue spaces analysed, which depend on their size. Consequently, the seasonal temperature fluctuations in larger lakes in which water volumes are expected to be larger are much smaller^7,20^. Our study found that, particularly in smaller lakes, lakes in general exhibit both surface thermal cooling and heating effects throughout the entire year. Due to their significant heat storage capacity, larger lakes are less affected by short warm or cold weather periods and, smaller lakes due to their lower heat storage capacity, respond faster to short-term weather fluctuations.

We found the greatest surface temperature cooling distances associated with larger lakes, underscoring their ability to influence thermal environment also across larger distances. Interestingly, three small lakes identified as outliers (0.2 to 0.3 ha) showed higher values for those distances. These small lakes are surrounded by extensive green spaces, suggesting a beneficial interplay between blue and green spaces. During the day, trees and vegetation can reduce water temperatures through shading, while at night they contribute to local cooling through cold-air production^4,5^. At the same time, urban vegetation typically warms more rapidly than water surfaces during daytime, leading to comparatively lower surface temperatures than their vegetated surroundings.

The greatest average surface cooling intensities were identified in our study for summer and spring (both 2.0 °C) (see^7^) confirming previous results for Asian case studies^11,16^. In spring, water surface temperatures remain low after the winter season, while surrounding areas are already warmer due to a lower heat storage capacity and increased sunshine duration. This temperature difference leads to high cooling intensities, especially for larger lakes.

The results of the Threshold Value of Efficiency (TVoE) are broadly consistent with the findings from Wuhan^27^, especially with regard to significantly increased values for the cooling distance compared to the cooling intensity^49^. In Hannover, the TVoE of 0.70 ha for cooling intensity during summer indicates that lakes of this size consistently show a surface cooling thermal effect while lakes with bigger areas do at least for our sample not lead to substantial additional benefits. Consequently, this threshold value could represent a locally context dependent reference but not a universally valid optimum. Previous studies have shown that the TVoE of blue and green spaces varies depending on the climate region and tends to increase with higher average annual temperatures and precipitation levels^49^. For example, a TVoE of 1.12 ha was reported for blue spaces in Copenhagen under a temperate oceanic climate^50^, while lower values were found for tropical monsoon climates, ranging from 0.45 to 0.70 ha in the Pearl River Delta metropolitan area, China^39^. The TVoE is, thus, case specific and depends very much on the local context conditions including local climate, urban morphology, and especially the size distribution of lakes^28^. High humidity for instance can strongly influence such threshold values, e.g. reducing the cooling effect in warm and humid climates or with contrasting effects in dry regions^51^.

For Hannover, we found that the land use classes Impervious areas and Bare Soil, Building Cover Ratio and High Vegetation are the most influential land use classes affecting the surface thermal characteristics of lakes, as was also shown in a case study for Baltimore, USA^18^. Impervious areas, and Building Cover Ratio, likewise, were shown to be positively associated with water surface temperature and heating intensity, especially during warmer months^52^. Distance to built-up areas emerged as an additional key factor being related to surface thermal effects. Significant negative associations with the indicators in most non-winter seasons indicate that surface temperatures of lakes located more distant from urbanized areas remain cooler and generate stronger and more extensive cooling, a pattern consistent with the surface urban heat island effect^1^. These findings are further supported by the hierarchical multivariate regression analysis, which highlights lake area and land cover structure indicators explaining the highest share of variance among the used indicators for cooling intensity, particularly in spring and summer (see Supplementary Tables 2–9). The explained variance is, overall, below 50% and even lower for cooling distance meaning that other factors not considered in this study also impact on water surface cooling (see discussion in limitations below).

These relationships between urban land use and surface thermal characteristics of lakes are related to the high heat storage capacity of impervious urban structures^53^ compared to natural landscapes, especially during the warmer months. High shares of vegetation consistently demonstrated significant negative associations with water surface temperature and positive impacts on increasing cooling intensity and distances, highlighting its moderating effect on water surface temperatures across seasons. We identified small lakes surrounded by large green spaces as outliers to show particularly long cooling distances. In particular, the shading^5^ and evaporative cooling^4^ of trees have a cooling effect on the adjacent water surface temperature and further on the surface cooling intensity and distance.

An additional indicator considered in or study was the Sky View Factor (SVF) which is regarded as an important parameter in studies of the urban microclimate as it reflects the degree of sky openness and is therefore closely linked to shading conditions and nocturnal radiative cooling processes^54^. In our study, however, the SVF showed no significant or non-consistent associations with the surface cooling effects caused by urban blue spaces. A key reason for this could be the lack of a clear distinction in SVF values between different land use types. Consequently, low SVF values in densely built-up areas may still be associated with relatively high land surface temperatures (LST), while high SVF values in green areas may coincide with comparatively low LST.

### Limitations of the approach

We acknowledge that our study focuses exclusively on blue spaces such as lakes and ponds, which are less thermally mixed compared to flowing water bodies and show thermal stratification^55^. This focus on lakes and ponds results in still waters exerting a different influence on air and surface temperatures than rivers, which exhibit more balanced temperatures^56^. Additionally, flowing waters tend to show lower cooling and heating intensities^7^.

Our study is based on remote sensing data recorded during different seasons between 10:15 and 10:20 a.m. UTC. At the study site in Hannover, Germany, this corresponds to 11:15–11:20 a.m. for the analysed data during winter season (CET) and 12:15–12:20 p.m. during spring, summer and autumn (CEST). This timing represents daytime conditions but does not match with the daily maximum air and surface temperatures, which typically occur in the afternoon, nor with the daily minimum temperatures observed just before sunrise^57^. Depending on the season, the recording time occurs a few hours after sunrise, resulting in surface temperatures that are generally elevated compared to the minimum temperatures. We also applied a multi-year and multi-season approach to improve robustness of results. However, our analysis is limited to three years and a limited number of available and useable satellite images and may, thus, be not entirely representative. The study also uses a single case study and results are – although comparable with findings from other studies as illustrated above – dependent on the local context^7,58,59^.

To assess the surface water effect of blue spaces, we used Landsat satellite data. The Landsat thermal band has a native spatial resolution of 100 m and is resampled to 30 m to match the multispectral bands. While this resampling facilitates spatial analysis, the thermal information remains constrained to the original pixel size and should be considered when interpreting fine-scale spatial patterns and results of gradient analyses. Consequently, satellite-derived LST provides a physically meaningful basis to assess and compare spatial patterns of lake-induced surface cooling effects, although the resulting cooling distances should be carefully interpretated.

We acknowledge that in this study, we address Land Surface Temperature (LST) which needs to be interpreted differently compared to air temperature^60^. Although LST and air temperature near the ground can correlate significantly, they are not directly comparable^61^. Surface urban heat island intensities derived from LST are generally stronger than Urban Heat Island estimates based on air temperature, as LST is strongly influenced by surface properties and radiation processes^61^. While spatial LST patterns may indicate surface variations in land use^62^, directly linking them to cooling effects in the urban atmosphere as in the context of human thermal comfort can be misleading. Nevertheless, LST remains a valuable indicator for assessing surface-based thermal effects because urban water bodies modify the local surface energy balance, creating temperature gradients between cooler water surfaces and the warmer built environment. Through local advection and turbulent mixing, these gradients reduce sensible heat flux and limit the heating of adjacent surfaces^15^. LST retrieved from thermal remote sensing captures these spatial patterns of surface temperature that result from differences in energy balance and heat fluxes.

We also acknowledge that the urban thermal environment is one aspect of the broader concept of microclimate, which also includes air humidity, wind speed and direction, and solar radiation—factors needed for assessing the thermal stress experienced by humans^63^. Our climatic background data (see supplementary material) suggested that relative humidity seems likely to be very much associated with the surface thermal effect indicators with higher cooling intensities, indicating that evaporation processes are mechanism driving the lake’s surface cooling capacity as mentioned by previous studies^51^. The climate background data available for this study originate, however, from only one weather station and primarily reflect general background conditions not allowing for in-depth statistical analyses. Local variations of climate background variables within the city are, however, very likely, especially in the case of near-ground wind fields, which can be strongly altered by the urban 3D-structure, e.g. buildings. We already included ground-level vegetation and high vegetation and also impervious areas, buildings and the sky view factor to integrate potential influences of height and openness. Future studies should consider not only mean building height but also the spatial distribution of building heights and building orientation with canyon structures to assess local wind patterns, shading conditions, and solar radiation exposure^64^.

Future research may consider, thus, multi-method approaches which combine field measurements of environmental parameters (air temperature, air humidity and wind speed) with urban structure indicators and remote sensing based analyses to accurately assess the full dimension of thermal dynamics and to better quantify “real” human heat stress and blue and green spaces cooling effect at pedestrian level^63^.

Characteristics of lakes or water bodies such as shape or depth were not assessed in this study but can play an important role in thermal effects. Shallow lakes usually have a lower volume leading to more efficient heat exchange^20^ and reduced cooling effects^65^. Lake area also impacts surface temperature and mixing, even in shallow lakes, as larger bodies of water can experience stronger wind-induced mixing, which can distribute heat more evenly throughout the water column^66,67^. Data on the water depth was not available for Hannover.

**Fig. 9:**
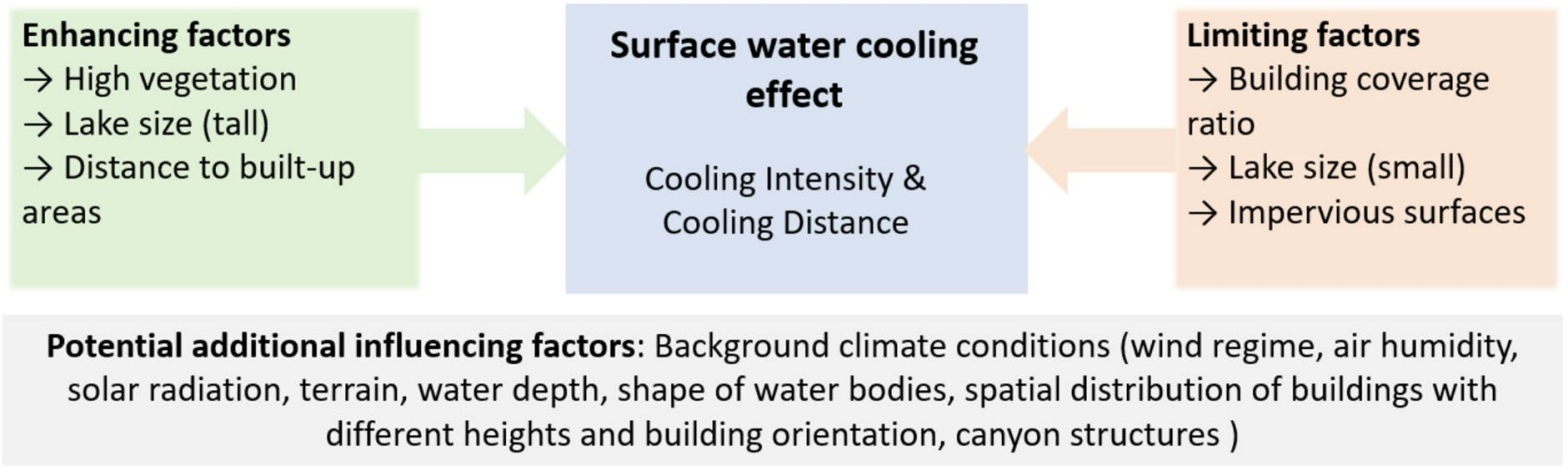
Conceptual summary of factors influencing the cooling effect of water bodies.

Figure 9 summarizes the main factors (potentially) influencing the thermal effect of water bodies as found in our study and those recommended to be included in future research.

## Conclusions

This study found that larger lakes provide the greatest surface temperature cooling intensity and distance for the case study city of Hannover particularly during summer, both in terms of intensity and spatial extent. The findings suggest that the cooling potential of blue spaces is linked to surrounding land cover characteristics. In particular, higher shares of vegetation, especially trees are associated with lower water surface temperatures and enhanced surface cooling effects, likely due to shading and evaporative cooling, which can help counterbalance potential heat generated by nearby urban infrastructure^58,68^. Immediate areas around lakes should if possible - limit impervious areas, such as asphalt and concrete, as these urban characteristics are associated with reduced cooling effectiveness and can counteract the thermal benefits provided by the lakes^52^. In densely built-up urban areas where integrating new blue spaces is challenging, alternative approaches such as the greening of blue space banks and surrounding areas and reducing impervious areas^68,69^ may be considered.

## Electronic Supplementary Material

Below is the link to the electronic supplementary material.

Supplementary Material 1

## Data Availability

The datasets generated during and/or analysed during the current study are available from the corresponding author on reasonable request.
