# Supplementary material for: Impact of urban blue spaces on urban surface temperatures - A seasonal perspective
Source: Sci Rep. 2026 May 9;16:14697. doi: 10.1038/s41598-026-49643-4 (PMC13157505; doi:10.1038/s41598-026-49643-4)
Supplement: Supplementary file 1 — Supplementary Material 1 [file 41598_2026_49643_MOESM1_ESM.docx]

**Supplementary material**

**Background climate conditions**

To qualitatively discuss a potential influence of the background climate condition on surface thermal effects of blue spaces, meteorological data from the closest German Weather Service (DWD) station for Hannover (the only one available with DWD), located at the border of the city were used. To align temporally with the day the remote sensing data were generated, 24-hour mean values of air temperature, relative humidity, and wind speed were calculated from the period preceding the Landsat satellite overpass. The 24-hour mean value was chosen to represent the integrated daily meteorological conditions. These data were used to describe the general context weather situation, while acknowledging that the very local urban conditions may deviate from the station measurements. No quantitative assessments could be done since data is limited to one station.

The data shows that relative humidity seems to be related to surface temperature cooling intensity values, with lower humidity levels consistently appearing when we recorded stronger surface cooling effects across all seasons (Supplementary Table 1). For instance, the strongest surface cooling ( 2.42 °C) occurred at a day with the lowest air humidity value (54.5%). A potential association between air temperature and surface cooling intensity is season-dependent and seemed non-uniform. A potential relation between wind speed and surface thermal effect indicators varies by season. For spring and summer, increased wind speeds were recorded the days when we found reduced surface cooling intensity, but no consistent trend is observed in autumn and winter.

| Supplementary Table 1: 24-hour meteorological parameters and lake cooling metrics | | | | | | |
| --- | --- | --- | --- | --- | --- | --- |
| **Season** | **Date** | **Air Temperature (°C)*** | **Mean relative humidity (%)*** | **Mean Wind Speed (m/s)*** | **Cooling Intensity (°C)** | **Cooling Distance (m)** |
| Winter | 20.12.2021 | -1.04 | 95.34 | 2.18 | 0.48 | 135.71 |
|  | 15.12.2022 | -4.21 | 83.2 | 1.78 | 0.23 | 152.08 |
|  | 27.01.2024 | 4.03 | 79.74 | 3.43 | -0.02 | 151.19 |
| Spring | 18.04.2022 | 9.8 | 54.65 | 2.3 | -2.00 | 316.67 |
|  | 30.04.2023 | 8.61 | 62.29 | 1.76 | -2.02 | 305.8 |
|  | 01.05.2024 | 19.31 | 60.97 | 4.61 | -1.85 | 302.9 |
| Summer | 24.07.2022 | 22.61 | 54.46 | 2.4 | -2.42 | 306.52 |
|  | 11.07.2023 | 23.79 | 58.07 | 3.35 | -1.87 | 288.81 |
|  | 13.08.2024 | 25.85 | 57.66 | 3.74 | -1.76 | 339.58 |
| Autumn | 02.09.2022 | 17.18 | 55.67 | 4.32 | -1.65 | 288.97 |
|  | 05.09.2023 | 21.57 | 63.86 | 2.51 | -1.70 | 323.13 |
|  | 25.10.2024 | 13.64 | 82.25 | 2.59 | -0.58 | 263.64 |
| *Note: Meteorological values represent 24-hour mean conditions prior to the overpass, based on data from the German Weather Service [41]. | | | | | | |

**Additional analyses on the relationship between urban morphological parameters and the surface cooling intensity and distance**

To assess the relative importance of lake size, land cover, and urban morphology, hierarchical multivariate regression models were applied for each year, season, and response variable (Surface Cooling Intensity and Surface Cooling Distance)(Supplementary Tables 2-9). Model 1 included lake area and land cover, Model 2 additionally incorporated SVF, building height, and urban distance, and Model 3 added the interaction between SVF and building height.

For cooling intensity (CI), Model 1 explained a substantial proportion of variance indicating that lake area and land cover are the dominant drivers shown particularly for spring and summer (up to ~40–43%), reflecting a stronger structural control of cooling under warmer conditions. Lake area showed consistent negative and often significant effects (e.g. p = 0.002–0.003), while high vegetation was also significant (p = 0.001–0.003). In contrast, building cover ratio showed positive and frequently significant effects (p ≤ 0.01), indicating reduced cooling in more built-up environments. Adding urban morphological variables (Model 2) resulted in moderate but inconsistent improvements, with the largest increases in summer (up to +7 % adjusted R²). However, SVF was not significant in any CI model, and building height and urban distance were only significant in isolated cases.

For cooling distance (CD), explanatory power was generally low and followed a similar seasonal pattern, with slightly higher values in spring and summer. Significant effects were mainly limited to high vegetation (p = 0.001–0.02) and, in selected cases, lake area, while additional predictors in Model 2 provided only minor gains.

The interaction term (Model 3) yielded limited additional explanatory value, with significant effects restricted to spring (p ≈ 0.015–0.023).

Overall, the results confirm that lake size and surrounding land cover composition explain the highest share of variance in the surface cooling effects, whereas urban morphological variables and their interactions play a secondary and context-dependent role, particularly under warmer seasonal conditions.

The explained variance is, overall, below 50% meaning that other factors not considered in this study impact on water surface cooling.

Supplementary Table 2: Results of hierarchical multivariate regression models for surface cooling intensity in spring.

|  |  | **β1** **(Lake area)** | **β2 (Impervious)** | **β3 (High vegetation)** | **β4 (Building Cover Ratio)** | **β5  (SVF)** | **β6  (Building height)** | **β7  (Urban distance)** | **β8 (SVF — Building height)** | **R2-adjusted (%)** |
| --- | --- | --- | --- | --- | --- | --- | --- | --- | --- | --- |
| 2022 | Model 1 | -0.595 | -0.350 | -0.477 | 0.587 |  |  |  |  | 28.3 |
|  | *p values* | ***0.002*** | *0.113* | ***0.039*** | ***0.005*** |  |  |  |  |  |
|  | Model 2 | -0.643 | -0.328 | -0.624 | 0.465 | -0.125 | 0.297 | 0.113 |  | 28.5 |
|  | *p values* | ***0.001*** | *0.152* | ***0.030*** | *0.062* | *0.638* | *0.164* | *0.626* |  |  |
|  | Model 3 | -0.656 | -0.336 | -0.659 | 0.395 | -0.051 | 0.611 | 0.174 | 0.483 | 33.2 |
|  | *p values* | ***0.001*** | *0.130* | ***0.019*** | *0.103* | *0.844* | ***0.014*** | *0.439* | ***0.017*** |  |
| 2023 | Model 1 | -0.391 | -0.231 | -0.791 | 0.578 |  |  |  |  | 30.2 |
|  | *p values* | *0.055* | *0.325* | ***0.002*** | ***0.010*** |  |  |  |  |  |
|  | Model 2 | -0.489 | -0.122 | -0.891 | 0.589 | -0.054 | 0.380 | 0.421 |  | 33.3 |
|  | *p values* | ***0.017*** | *0.608* | ***0.003*** | ***0.025*** | *0.846* | *0.088* | *0.083* |  |  |
|  | Model 3 | -0.494 | -0.125 | -0.904 | 0.563 | -0.026 | 0.498 | 0.444 | 0.180 | 33.0 |
|  | *p values* | ***0.017*** | *0.600* | ***0.003*** | ***0.033*** | *0.926* | *0.061* | *0.071* | *0.403* |  |
| 2024 | Model 1 | -0.471 | -0.401 | -0.505 | 0.738 |  |  |  |  | 39.8 |
|  | *p values* | ***0.003*** | ***0.029*** | ***0.009*** | ***0.000*** |  |  |  |  |  |
|  | Model 2 | -0.519 | -0.344 | -0.581 | 0.752 | -0.094 | 0.105 | 0.232 |  | 39.0 |
|  | *p values* | ***0.002*** | *0.071* | ***0.016*** | ***0.000*** | *0.670* | *0.551* | *0.229* |  |  |
|  | Model 3 | -0.530 | -0.350 | -0.609 | 0.696 | -0.035 | 0.355 | 0.280 | 0.384 | 42.6 |
|  | *p values* | ***0.001*** | *0.059* | ***0.009*** | ***0.001*** | *0.871* | *0.082* | *0.137* | ***0.023*** |  |

| Supplementary Table 3: Results of hierarchical multivariate regression models for surface cooling intensity in summer. | | | | | | | | | | |
| --- | --- | --- | --- | --- | --- | --- | --- | --- | --- | --- |
|  |  | **β1** **(Lake area)** | **β2 (Impervious)** | **β3 (High vegetation)** | **β4 (Building Cover Ratio)** | **β5  (SVF)** | **β6  (Building height)** | **β7  (Urban distance)** | **β8 (SVF — Building height)** | **R2-adjusted (%)** |
| 2022 | Model 1 | -0.529 | -0.338 | -0.948 | 0.405 |  |  |  |  | 28.7 |
|  | *p values* | ***0.018*** | *0.185* | ***0.001*** | *0.090* |  |  |  |  |  |
|  | Model 2 | -0.622 | -0.236 | -1.082 | 0.407 | -0.13 | 0.294 | 0.409 |  | 29.9 |
|  | *p values* | ***0.006*** | *0.368* | ***0.001*** | *0.154* | *0.670* | *0.230* | *0.126* |  |  |
|  | Model 3 | -0.621 | -0.236 | -1.081 | 0.409 | -0.132 | 0.286 | 0.408 | -0.012 | 28.9 |
|  | *p values* | ***0.007*** | *0.372* | ***0.002*** | *0.158* | *0.670* | *0.325* | *0.133* | *0.961* |  |
| 2023 | Model 1 | -0.380 | -0.018 | -0.566 | 0.531 |  |  |  |  | 30.3 |
|  | *p values* | ***0.034*** | *0.930* | ***0.009*** | ***0.007*** |  |  |  |  |  |
|  | Model 2 | -0.494 | 0.096 | -0.762 | 0.490 | -0.189 | 0.405 | 0.469 |  | 37.6 |
|  | *p values* | ***0.005*** | *0.632* | ***0.003*** | ***0.027*** | *0.422* | ***0.033*** | ***0.024*** |  |  |
|  | Model 3 | -0.501 | 0.093 | -0.779 | 0.455 | -0.152 | 0.561 | 0.499 | 0.239 | 38.3 |
|  | *p values* | ***0.004*** | *0.644* | ***0.003*** | ***0.041*** | *0.518* | ***0.013*** | ***0.017*** | *0.189* |  |
| 2024 | Model 1 | -0.378 | -0.012 | -0.205 | 0.560 |  |  |  |  | 28.8 |
|  | *p values* | ***0.009*** | *0.943* | *0.234* | ***0.000*** |  |  |  |  |  |
|  | Model 2 | -0.454 | 0.068 | -0.335 | 0.542 | -0.131 | 0.256 | 0.326 |  | 32.9 |
|  | *p values* | ***0.002*** | *0.682* | *0.109* | ***0.004*** | *0.502* | *0.101* | *0.056* |  |  |
|  | Model 3 | -0.462 | 0.064 | -0.354 | 0.503 | -0.089 | 0.430 | 0.361 | 0.269 | 35.0 |
|  | *p values* | ***0.001*** | *0.696* | *0.086* | ***0.006*** | *0.642* | ***0.019*** | ***0.034*** | *0.072* |  |

| Supplementary Table 4: Results of hierarchical multivariate regression models for surface cooling intensity in autumn. | | | | | | | | | | |
| --- | --- | --- | --- | --- | --- | --- | --- | --- | --- | --- |
|  |  | **β1** **(Lake area)** | **β2 (Impervious)** | **β3 (High vegetation)** | **β4 (Building Cover Ratio)** | **β5  (SVF)** | **β6  (Building height)** | **β7  (Urban distance)** | **β8 (SVF — Building height)** | **R2-adjusted (%)** |
| 2022 | Model 1 | -0.215 | -0.044 | -0.318 | 0.349 |  |  |  |  | 17.3 |
|  | *p values* | *0.131* | *0.789* | *0.066* | ***0.025*** |  |  |  |  |  |
|  | Model 2 | -0.262 | -0.001 | -0.385 | 0.320 | -0.038 | 0.223 | 0.171 |  | 17.7 |
|  | *p values* | *0.074* | *0.995* | *0.073* | *0.086* | *0.847* | *0.163* | *0.324* |  |  |
|  | Model 3 | -0.263 | -0.002 | -0.389 | 0.311 | -0.029 | 0.262 | 0.179 | 0.061 | 16.7 |
|  | *p values* | *0.074* | *0.991* | *0.072* | *0.099* | *0.886* | *0.167* | *0.309* | *0.693* |  |
| 2023 | Model 1 | -0.466 | -0.116 | -0.321 | 0.326 |  |  |  |  | 20.7 |
|  | *p values* | ***0.004*** | *0.522* | *0.092* | *0.057* |  |  |  |  |  |
|  | Model 2 | -0.539 | -0.018 | -0.391 | 0.395 | -0.084 | 0.144 | 0.382 |  | 22.7 |
|  | *p values* | ***0.001*** | *0.923* | *0.095* | *0.054* | *0.700* | *0.406* | ***0.046*** |  |  |
|  | Model 3 | -0.545 | -0.021 | -0.404 | 0.368 | -0.055 | 0.265 | 0.406 | 0.186 | 23.0 |
|  | *p values* | ***0.001*** | *0.910* | *0.084* | *0.073* | *0.800* | *0.197* | ***0.035*** | *0.270* |  |
| 2024 | Model 1 | -0.220 | -0.072 | -0.165 | 0.125 |  |  |  |  | 33.9 |
|  | *p values* | ***0.000*** | *0.242* | ***0.011*** | ***0.032*** |  |  |  |  |  |
|  | Model 2 | -0.234 | -0.063 | -0.198 | 0.104 | -0.027 | 0.070 | 0.042 |  | 33.4 |
|  | *p values* | ***0.000*** | *0.330* | ***0.015*** | *0.138* | *0.716* | *0.240* | *0.516* |  |  |
|  | Model 3 | -0.233 | -0.062 | -0.196 | 0.108 | -0.031 | 0.055 | 0.039 | -0.024 | 32.6 |
|  | *p values* | ***0.000*** | *0.336* | ***0.017*** | *0.131* | *0.684* | *0.441* | *0.552* | *0.681* |  |

| Supplementary Table 5: Results of hierarchical multivariate regression models for surface cooling intensity in winter. | | | | | | | | | | |
| --- | --- | --- | --- | --- | --- | --- | --- | --- | --- | --- |
|  |  | **β1** **(Lake area)** | **β2 (Impervious)** | **β3 (High vegetation)** | **β4 (Building Cover Ratio)** | **β5  (SVF)** | **β6  (Building height)** | **β7  (Urban distance)** | **β8 (SVF — Building height)** | **R2-adjusted (%)** |
| 2022 | Model 1 | 0.298 | 0.17 | 0.029 | -0.115 |  |  |  |  | 29.9 |
|  | *p values* | ***0.000*** | ***0.018*** | *0.695* | *0.084* |  |  |  |  |  |
|  | Model 2 | 0.273 | 0.191 | -0.028 | -0.141 | -0.056 | 0.104 | 0.092 |  | 31.9 |
|  | *p values* | ***0.000*** | ***0.010*** | *0.753* | *0.075* | *0.504* | *0.125* | *0.213* |  |  |
|  | Model 3 | 0.276 | 0.193 | -0.021 | -0.126 | -0.072 | 0.037 | 0.079 | -0.102 | 33.3 |
|  | *p values* | ***0.000*** | ***0.008*** | *0.815* | *0.109* | *0.393* | *0.633* | *0.282* | *0.117* |  |
| 2023 | Model 1 | 0.021 | 0.246 | 0.003 | -0.18 |  |  |  |  | 18.1 |
|  | *p values* | *0.716* | ***0.000*** | *0.962* | ***0.004*** |  |  |  |  |  |
|  | Model 2 | 0.009 | 0.252 | -0.04 | -0.208 | -0.046 | 0.058 | 0.034 |  | 16.8 |
|  | *p values* | *0.884* | ***0.000*** | *0.645* | ***0.007*** | *0.568* | *0.368* | *0.623* |  |  |
|  | Model 3 | 0.012 | 0.254 | -0.03 | -0.19 | -0.065 | -0.024 | 0.018 | -0.126 | 20.6 |
|  | *p values* | *0.833* | ***0.000*** | *0.718* | ***0.012*** | *0.41* | *0.745* | *0.79* | ***0.041*** |  |
| 2024 | Model 1 | -0.164 | 0.051 | 0.011 | -0.018 |  |  |  |  | 12.1 |
|  | *p values* | ***0.000*** | *0.323* | *0.834* | *0.714* |  |  |  |  |  |
|  | Model 2 | -0.154 | 0.014 | -0.076 | -0.129 | -0.109 | 0.031 | -0.114 |  | 22.9 |
|  | *p values* | ***0.001*** | *0.775* | *0.225* | ***0.021*** | *0.068* | *0.504* | ***0.028*** |  |  |
|  | Model 3 | -0.153 | 0.015 | -0.074 | -0.124 | -0.113 | 0.012 | -0.118 | -0.029 | 22.3 |
|  | *p values* | ***0.001*** | *0.769* | *0.241* | ***0.027*** | ***0.060*** | *0.825* | ***0.025*** | *0.522* |  |

| Supplementary Table 6: Results of hierarchical multivariate regression models for surface cooling distance in spring. | | | | | | | | | | |
| --- | --- | --- | --- | --- | --- | --- | --- | --- | --- | --- |
|  |  | **β1** **(Lake area)** | **β2 (Impervious)** | **β3 (High vegetation)** | **β4 (Building Cover Ratio)** | **β5  (SVF)** | **β6  (Building height)** | **β7  (Urban distance)** | **β8 (SVF — Building height)** | **R2-adjusted (%)** |
| 2022 | Model 1 | -11.149 | -19.612 | -50.535 | 55.189 |  |  |  |  | 7.0 |
|  | *p values* | *0.685* | *0.539* | *0.131* | *0.066* |  |  |  |  |  |
|  | Model 2 | -15.671 | -19.753 | -67.128 | 35.109 | -9.814 | 44.424 | 2.369 |  | 6.8 |
|  | *p values* | *0.579* | *0.551* | *0.107* | *0.331* | *0.800* | *0.153* | *0.944* |  |  |
|  | Model 3 | -17.658 | -20.899 | -72.369 | 24.628 | 1.217 | 91.129 | 11.498 | 71.833 | 13.2 |
|  | *p values* | *0.517* | *0.514* | *0.073* | *0.482* | *0.974* | ***0.011*** | *0.725* | ***0.015*** |  |
| 2023 | Model 1 | -48.928 | -2.525 | -90.856 | 29.107 |  |  |  |  | 17.6 |
|  | *p values* | *0.086* | *0.938* | ***0.009*** | *0.341* |  |  |  |  |  |
|  | Model 2 | -53.97 | -0.587 | -101.81 | 15.551 | -2.468 | 43.244 | 8.59 |  | 16.8 |
|  | *p values* | *0.066* | *0.986* | ***0.019*** | *0.675* | *0.951* | *0.177* | *0.804* |  |  |
|  | Model 3 | -54.105 | -0.665 | -102.164 | 14.842 | -1.722 | 46.404 | 9.208 | 4.86 | 15.6 |
|  | *p values* | *0.067* | *0.985* | ***0.020*** | *0.693* | *0.966* | *0.223* | *0.793* | *0.876* |  |
| 2024 | Model 1 | -2.999 | -26.428 | -54.862 | 98.786 |  |  |  |  | 19.1 |
|  | *p values* | *0.910* | *0.390* | *0.089* | ***0.001*** |  |  |  |  |  |
|  | Model 2 | 0.276 | -33.035 | -68.395 | 84.804 | -22.869 | -14.438 | -20.268 |  | 16.9 |
|  | *p values* | *0.992* | *0.308* | *0.093* | ***0.018*** | *0.545* | *0.632* | *0.537* |  |  |
|  | Model 3 | -1.61 | -34.124 | -73.369 | 74.857 | -12.399 | 29.89 | -11.604 | 68.178 | 22.2 |
|  | *p values* | *0.952* | *0.277* | *0.063* | ***0.031*** | *0.736* | *0.387* | *0.716* | ***0.018*** |  |

| Supplementary Table 7: Results of hierarchical multivariate regression models for surface cooling distance in summer. | | | | | | | | | | |
| --- | --- | --- | --- | --- | --- | --- | --- | --- | --- | --- |
|  |  | **β1** **(Lake area)** | **β2 (Impervious)** | **β3 (High vegetation)** | **β4 (Building Cover Ratio)** | **β5  (SVF)** | **β6  (Building height)** | **β7  (Urban distance)** | **β8 (SVF — Building height)** | **R2-adjusted (%)** |
| 2022 | Model 1 | -6.739 | -15.464 | -109.73 | 9.565 |  |  |  |  | 16.5 |
|  | *p values* | *0.791* | *0.599* | ***0.001*** | *0.727* |  |  |  |  |  |
|  | Model 2 | -18.868 | 1.192 | -112.127 | 24.273 | 2.945 | 34.415 | 61.738 |  | 19.4 |
|  | *p values* | *0.461* | *0.968* | ***0.004*** | *0.457* | *0.933* | *0.221* | ***0.046*** |  |  |
|  | Model 3 | -18.802 | 1.23 | -111.953 | 24.62 | 2.579 | 32.867 | 61.435 | -2.38 | 18.3 |
|  | *p values* | *0.465* | *0.968* | ***0.004*** | *0.457* | *0.942* | *0.325* | *0.050* | *0.931* |  |
| 2023 | Model 1 | -13.241 | 34.887 | -74.869 | 31.846 |  |  |  |  | 21.9 |
|  | *p values* | *0.580* | *0.210* | ***0.011*** | *0.220* |  |  |  |  |  |
|  | Model 2 | -25.029 | 48.503 | -85.695 | 35.422 | -5.953 | 42.524 | 52.415 |  | 25.3 |
|  | *p values* | *0.297* | *0.088* | ***0.017*** | *0.248* | *0.856* | *0.108* | *0.070* |  |  |
|  | Model 3 | -25.796 | 48.061 | -87.716 | 31.379 | -1.699 | 60.537 | 55.935 | 27.705 | 25.5 |
|  | *p values* | *0.282* | *0.090* | ***0.014*** | *0.309* | *0.959* | *0.054* | *0.054* | *0.277* |  |
| 2024 | Model 1 | -50.352 | 67.231 | -4.848 | 64.083 |  |  |  |  | 16.7 |
|  | *p values* | *0.087* | ***0.049*** | *0.890* | ***0.045*** |  |  |  |  |  |
|  | Model 2 | -59.953 | 74.358 | -29.247 | 50.068 | -23.568 | 44.242 | 31.913 |  | 17.5 |
|  | *p values* | ***0.046*** | ***0.036*** | *0.502* | *0.189* | *0.564* | *0.176* | *0.369* |  |  |
|  | Model 3 | -60.571 | 74.001 | -30.876 | 46.809 | -20.138 | 58.762 | 34.751 | 22.332 | 16.9 |
|  | *p values* | ***0.045*** | ***0.037*** | *0.481* | *0.224* | *0.625* | *0.131* | *0.333* | *0.481* |  |

| Supplementary Table 8: Results of hierarchical multivariate regression models for surface cooling distance in autumn. | | | | | | | | | | |
| --- | --- | --- | --- | --- | --- | --- | --- | --- | --- | --- |
|  |  | **β1** **(Lake area)** | **β2 (Impervious)** | **β3 (High vegetation)** | **β4 (Building Cover Ratio)** | **β5  (SVF)** | **β6  (Building height)** | **β7  (Urban distance)** | **β8 (SVF — Building height)** | **R2-adjusted (%)** |
| 2022 | Model 1 | -6.255 | -1.509 | -39.596 | 32.807 |  |  |  |  | 2.9 |
|  | *p values* | *0.805* | *0.959* | *0.198* | *0.233* |  |  |  |  |  |
|  | Model 2 | -15.601 | 5.256 | -65.77 | 18.129 | -27.345 | 40.45 | 31.229 |  | 4.9 |
|  | *p values* | *0.544* | *0.862* | *0.084* | *0.580* | *0.439* | *0.153* | *0.310* |  |  |
|  | Model 3 | -15.655 | 5.225 | -65.915 | 17.839 | -27.04 | 41.738 | 31.481 | 1.982 | 3.6 |
|  | *p values* | *0.545* | *0.863* | *0.086* | *0.592* | *0.451* | *0.215* | *0.312* | *0.942* |  |
| 2023 | Model 1 | -44.99 | 30.403 | -56.011 | -3.475 |  |  |  |  | 10.5 |
|  | *p values* | *0.090* | *0.320* | *0.081* | *0.903* |  |  |  |  |  |
|  | Model 2 | -48.891 | 38.907 | -49.962 | 13.762 | 4.377 | -6.269 | 30.438 |  | 8.2 |
|  | *p values* | *0.076* | *0.227* | *0.214* | *0.693* | *0.907* | *0.834* | *0.351* |  |  |
|  | Model 3 | -49.631 | 38.48 | -51.915 | 9.858 | 8.486 | 11.13 | 33.839 | 26.76 | 8.0 |
|  | *p values* | *0.072* | *0.233* | *0.198* | *0.779* | *0.822* | *0.753* | *0.304* | *0.358* |  |
| 2024 | Model 1 | -9.404 | -0.598 | -89.297 | 12.198 |  |  |  |  | 17.7 |
|  | *p values* | *0.669* | *0.981* | ***0.001*** | *0.608* |  |  |  |  |  |
|  | Model 2 | -19.531 | 11.568 | -91.33 | 18.193 | 8.26 | 44.854 | 44.331 |  | 21.3 |
|  | *p values* | *0.375* | *0.654* | ***0.006*** | *0.517* | *0.785* | *0.066* | *0.094* |  |  |
|  | Model 3 | -18.601 | 12.105 | -88.877 | 23.101 | 3.095 | 22.985 | 40.057 | -33.634 | 22.5 |
|  | *p values* | *0.395* | *0.637* | ***0.007*** | *0.411* | *0.918* | *0.417* | *0.129* | *0.150* |  |

| Supplementary Table 9: Results of hierarchical multivariate regression models for surface cooling distance in winter. | | | | | | | | | | |
| --- | --- | --- | --- | --- | --- | --- | --- | --- | --- | --- |
|  |  | **β1** **(Lake area)** | **β2 (Impervious)** | **β3 (High vegetation)** | **β4 (Building Cover Ratio)** | **β5  (SVF)** | **β6  (Building height)** | **β7  (Urban distance)** | **β8 (SVF — Building height)** | **R2-adjusted (%)** |
| 2022 | Model 1 | 29.334 | 27.811 | -19.208 | -26.658 |  |  |  |  | 3.6 |
|  | *p values* | *0.127* | *0.211* | *0.405* | *0.199* |  |  |  |  |  |
|  | Model 2 | 26.714 | 29.482 | -20.196 | -30.51 | 6.16 | 24.51 | 5.509 |  | 1.4 |
|  | *p values* | *0.179* | *0.206* | *0.486* | *0.228* | *0.821* | *0.259* | *0.815* |  |  |
|  | Model 3 | 27.465 | 29.916 | -18.216 | -26.548 | 1.991 | 6.859 | 2.059 | -27.148 | 2.4 |
|  | *p values* | *0.165* | *0.198* | *0.528* | *0.295* | *0.942* | *0.788* | *0.93* | *0.196* |  |
| 2023 | Model 1 | 1.008 | 38.678 | 20.671 | -42.929 |  |  |  |  | 1.6 |
|  | *p values* | *0.965* | *0.147* | *0.454* | *0.085* |  |  |  |  |  |
|  | Model 2 | 2.458 | 31.321 | 2.969 | -67.084 | -19.295 | 14.332 | -23.106 |  | 0.4 |
|  | *p values* | *0.917* | *0.260* | *0.931* | ***0.028*** | *0.551* | *0.579* | *0.412* |  |  |
|  | Model 3 | 3.18 | 31.738 | 4.872 | -63.278 | -23.3 | -2.628 | -26.421 | -26.084 | 0.5 |
|  | *p values* | *0.893* | *0.253* | *0.888* | ***0.039*** | *0.475* | *0.931* | *0.351* | *0.298* |  |
| 2024 | Model 1 | -63.098 | 3.323 | 4.949 | 2.686 |  |  |  |  | 9.0 |
|  | *p values* | ***0.001*** | *0.879* | *0.828* | *0.895* |  |  |  |  |  |
|  | Model 2 | -58.986 | -10.011 | -19.941 | -34.379 | -27.991 | 14.891 | -43.487 |  | 14.9 |
|  | *p values* | ***0.002*** | *0.649* | *0.468* | *0.153* | *0.278* | *0.468* | ***0.054*** |  |  |
|  | Model 3 | -58.148 | -9.528 | -17.731 | -29.96 | -32.642 | -4.801 | -47.336 | -30.286 | 16.5 |
|  | *p values* | ***0.002*** | *0.662* | *0.515* | *0.211* | *0.206* | *0.841* | ***0.036*** | *0.127* |  |

5
